# Incidence of Nd:YAG laser capsulotomy following cataract surgery: a population-based nation-wide study – FreYAG1 study

**DOI:** 10.1186/s12886-023-03134-6

**Published:** 2023-10-16

**Authors:** Antoine P. Brézin, Antoine Labbe, Cédric Schweitzer, François Lignereux, Pascal Rozot, Mélanie Goguillot, Françoise Bugnard, Corinne Dot

**Affiliations:** 1 Department of Ophthalmology, Université Paris Cité, Cochin Hospital, APHP, 27 rue du faubourg Saint-Jacques, Paris, 75014 France; 2 Department of Ophthalmology, Quinze-Vingts National Ophthalmology Hospital, Paris, France; 3 Department of Ophthalmology, CHU Bordeaux, Univ. Bordeaux, ISPED, INSERM, U1219 – Bordeaux Population Health Research Centre, Bordeaux, F-33000 France; 4 Department of Ophthalmology Santé Atlantique Polyclinic, Nantes, France; 5 Department of Ophthalmology, Juge Clinic, Marseille, France; 6 stève Consultants, Oullins, France; 7 Department of Ophthalmology, Desgenettes Military Hospital, Lyon, France

**Keywords:** Ophthalmology, Cataract, Nd:YAG, Dataclaims, Epidemiology

## Abstract

**Rationale:**

Nd:YAG (neodymium:yttrium-aluminum-garnet) capsulotomy (*Nd:YAG-caps*) is the gold standard for the treatment of PCO (Posterior Capsule Opacification). There is a lack of real-world data about *Nd:YAG-caps* use.

**Purpose:**

This study’s objectives were to estimate *Nd:YAG-caps* incidence in France, to describe the patient characteristics, and to analyze the time between surgeries and capsulotomies. Setting: The study was based on data extracted from the EGB database, a 1/97th sample representative of the French population. Design: observational, retrospective, cohort study using national claims data.

**Methods:**

French adult patients who underwent *Nd:YAG-caps* between 2014 and 2017 were selected. Main outcomes were the number of patients and procedures performed and the risk factors associated with early *Nd:YAG-caps*. Results: During the study period, *Nd:YAG-caps* were performed in 8,425 patients accounting for 10,774 procedures. The extrapolation to the French population led to estimate that 253.10^3^ patients had *Nd:YAG-caps*, representing 312.10^3^ procedures in 2017. The mean age at *Nd:YAG-caps* was 75.1 (± 10.2) years. About 36% of patients presented at least one ocular comorbidity. *Nd:YAG-caps* was performed within 2 years after surgery in 33.0% of patients and within one year in 9.8% of patients. Patients with *Nd:YAG-caps* within the first year (OR CI95 0.721 [0.673–0.772]) or in the first two years (OR CI95 0.721 [0.673–0.772]) were younger than patients with later *Nd:YAG-caps* and had a more frequent history of treated ocular diseases (OR 1.516 and 1.178, respectively).

**Conclusions:**

This study brought new real-world and large-scale data regarding *Nd:YAG-caps* use and gave an updated insight into the patients’ characteristics.

**Supplementary Information:**

The online version contains supplementary material available at 10.1186/s12886-023-03134-6.

## Introduction

More than 940.10^3^ cataract surgeries were performed in France in 2021, making cataract surgery the most frequent ophthalmological procedure [1, 2]. Secondary posterior capsular opacification (PCO) is a common complication, occurring within months after surgery, with an estimated 5-year rate between 30% and 50% [3–5]. PCO is managed by performing a capsulotomy with a Nd:YAG (neodymium:yttrium-aluminum-garnet) laser [6], allowing patients to regain satisfying vision within hours. Post-operative treatment can include anti-inflammatory and/or hypotonizing eyedrops for several days [7, 8].

With a significant proportion of cataract surgeries ultimately resulting in treated PCO, these ophthalmic procedures represent a substantial economic burden and public health impact. In addition, other potential adverse events due to cataract surgery or capsulotomy may increase this burden and deteriorate patients’ quality of life, in a population mostly consisting of elderly and comorbid patients [9, 10]. Recent and exhaustive knowledge on the most frequent complication of cataract surgery, as well as on the potential factors influencing its occurrence, is crucial for health authorities decision-making, even more with the global aging of the population.

There is a lack of real-world data about the epidemiology of Nd:YAG capsulotomy (*Nd:YAG-caps*). Few databases allow to access patient-level data with both sufficient representativeness and data granularity to ensure results generalizability. The French national representative sample (*Echantillon Généraliste des Bénéficiaires* – EGB), satisfies both conditions and exhaustively captures patients’ reimbursed healthcare resources and can complement the clinical studies carried on smaller populations, notably focusing on the risk of Nd:YAG-caps by type of implant. This FreYAG1 study aimed at estimating the up-to-date number of *Nd:YAG-caps* performed in France, describing patients’ characteristics, analyzing the delay between surgery and capsulotomy, and assessing risk factors of earlier YAG, notably regarding age.

## Materials and methods

### General design

This was an observational, retrospective, cohort study performed among patients who underwent *Nd:YAG-caps* between January 1st, 2014, and December 31st, 2017. This study used reimbursement data from the EGB, based on healthcare claims from the French national health data system.

### Study population

Adults who underwent *Nd:YAG-caps* between January 1st, 2014, and December 31st, 2017 (study period), were identified in EGB to assess overall and annual use of *Nd:YAG-caps*. Among them, patients with complete coverage (continuous affiliation to an insurance scheme) from January 1st, 2012, were considered for characteristics description. *Nd:YAG-caps* were identified using a specific code from French procedure classification. The date of *Nd:YAG-caps* was defined as the index date. In case of multiple *Nd:YAG-caps*, the first one was considered. Comorbidities and medical history of interest were assessed over a 2-year period before index date.

### Data source

The EGB is a 1/97th sample of insured individuals and gathers ≈ 700.10^3^ persons. It is an exhaustive pseudonymized patient-level collection of claims data, representative of the French population in terms of age, gender, and geographical area [11–13]. It exhaustively gathers patients’ healthcare reimbursements, using specific coding systems for procedures, laboratory tests, medical devices, diagnoses (hospitalizations), or drugs [14–17]. Only expensive drugs and medical devices are captured during hospitalizations, as others are part of the hospital stay fee (Diagnosis Related Groups [DRG]). Beneficiary data include age, gender, city of residence, date of care, care settings, as well as date and cause of death.

### Outcomes

The primary outcome was the number of patients undergoing *Nd:YAG-caps* and the number of procedures performed. They have been estimated overall and by year over study period. Data from EGB were extrapolated to the national scale for each year, adjusted on age and gender based on the French population census for the same year [18].

The secondary outcomes were the description of the patients’ characteristics. The sociodemographic characteristics were analyzed at index date and included age, gender, and district of residence. The comorbidities were assessed using validated algorithms based on hospital diagnoses and outpatient reimbursed treatments and were split into 3 categories. The non-ocular comorbidities included cardiovascular diseases, diabetes, malignant neoplasms, assessed over a 2-year period before index date. The ocular comorbidities included retinal vein occlusion (RVO), uveitis, diabetic retinopathy, prior *Nd:YAG-caps*, and cataract surgery, assessed over the same period. Finally, known *Nd:YAG-caps* complications were analyzed over a 1-year period before the index date and included Ocular hypertension (OHT) or glaucoma, retinal detachment (RD), vitrectomy, and treated diabetic macular edema (DME), age-related macular degeneration (ARMD), uveitis, or RVO [19]. Detailed algorithms are available as supplementary material. Patients’ characteristics were also analyzed according to the time between surgery and *Nd:YAG-caps*, which was categorized as very early (≤ 1 year post-surgery), early (between 1 and 2 years post-surgery), and late (> 2 years post-surgery).

### Statistical methods

All analyses were performed using the SAS® version 9.4 (SAS Institute Inc. Cary, NC, USA). Quantitative variables were described as means, standard deviations, medians, first (Q1) and third (Q3) quartiles, and extreme values; qualitative variables as absolute frequencies and percentages by category. Logistic regression models were developed including characteristics of interest, using a backward method with preliminary selection. Interactions with a p-value threshold ≤ 0.10 were included in the final models. The covariables of interest were the age at the index date; gender; ocular and non-ocular comorbidities, as well as known *Nd:YAG-caps* complications.

### Ethical considerations

Prior to data management, access to SNDS data was granted by the French national health data institute (*Institut National des Données de Santé* – INDS), implying the protocol validation by the French expert committee for health research and evaluation (*Comité d’Expertise pour les Recherches, les Études et les Évaluations dans le domaine de la Santé* - CEREES), as well as by the French national data protection agency (*Commission Nationale de l’Informatique et des Libertés* – CNIL). Once approved, EGB data were analyzed on the SNDS secure portal by data managers and statisticians trained to patient-related data securization. No individual data was extracted from the SNDS portal. This study was conducted in compliance with the French Data Protection Act and in accordance with applicable ethical principles set out in the Declaration of Helsinki.

## Results

### Nd:YAG laser capsulotomy

**Fig. 1:**
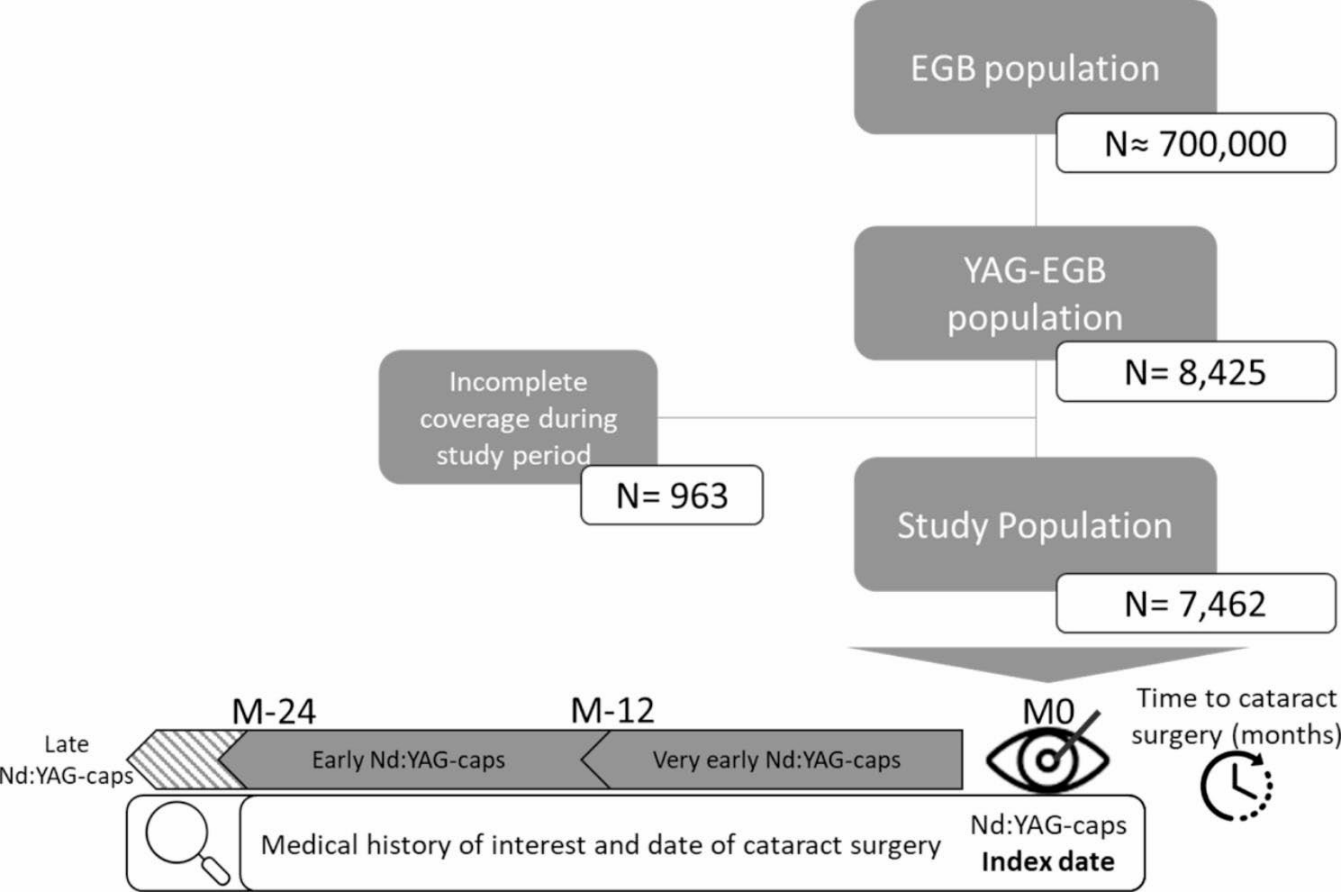
Patient Disposition over study period (January 1st, 2014 – December 31st, 2017)

During the study period, 8,425 patients underwent *Nd:YAG-caps*, accounting for 10,774 procedures, as bilateral capsulotomy can occur (Fig. 1).

**Fig. 2:**
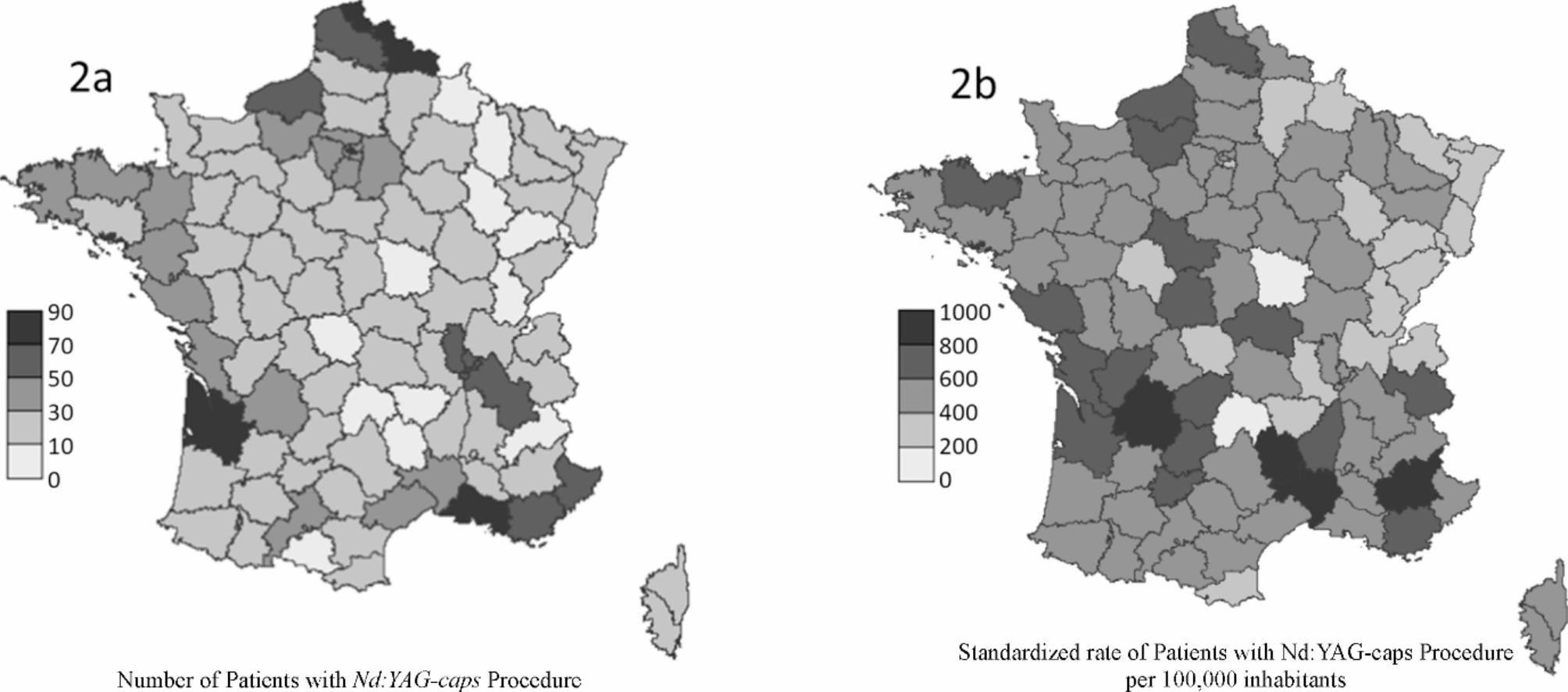
Number of Patients (**a**) and Standardized rate per 100,000 inhabitants (**b**) of Patients with *Nd:YAG-caps* Procedure – Year 2017 (N = 2,550)

More than 2,000 patients had *Nd:YAG-caps* each year, ranging from 2,006 (2,312 procedures) in 2014 to 2,550 (3,141 procedures) in 2017. In 2017, the EGB extrapolation [CI95%] to the French population estimated that 253.10^3^ [252.10^3^; 254.10^3^] patients had a *Nd:YAG-caps*, corresponding to 312.10^3^ [311.10^3^; 313.10^3^] procedures. In 2017, 9 districts (out of 100) accounted for > 20% of patients with *Nd:YAG-caps*. Among them, Gironde (n = 282), Bouches-du-Rhône (n = 269) and Nord (n = 257) each represented > 3% of overall patients (Fig. 2). For the same year, 4 French districts had > 800 *Nd:YAG-caps* performed per 100,000 inhabitants, all of them in the south of France (Table 1 – Fig. 2).

**Table 1:** *Nd:YAG-caps* Extrapolation to General Population

	Patients			Procedures		
**Year**	EGB data Patients n	Extrapolation Patients in thousands n [CI95%]	Change from 2014 (%)	EGB data Procedures n	Extrapolation Procedures in thousands n [CI95%]	Change from 2014 (%)
**2014**	2,006	227 [226 ; 228]	/	2,312	262 [261 ; 263]	/
**2015**	2,191	248 [247 ; 249]	+ 9.3%	2,550	289 [288 ; 290]	+ 10.4%
**2016**	2,306	229 [228 ; 230]	+ 0.9%	2,771	275 [274 ; 276]	+ 5.2%
**2017**	2,550	253 [252 ; 254]	+ 11.5%	3,141	312 [311 ; 313]	+ 19.2%

### Patient characteristics

Among the 8,425 patients with a *Nd:YAG-caps*, 7,462 patients (9,560 procedures) had no discontinuation in healthcare coverage. The sex ratio was 0.55 with 4,817 (64.6%) women. The mean (SD) age at index date was 75.1 (10.2) years, with most patients being aged ≥ 75 years (n = 4,409; 59.1%), while patients aged < 40 years accounted for 0.6% (Table 2).

**Table 2:** Patient Demographic and Clinical Characteristics at Index Date

Characteristics	*Nd:YAG-caps* patients (N = 7,462)
Gender, n (%)	
Male	2,645 (35.4%)
Female	4,817 (64.6%)
Sex-ratio	0.55
**Age, years, mean (sd)**	75.1 (10.2)
**Age, classes, n (%)**	
[18 ; 40[	42 (0.6%)
[40 ; 55[	272 (3.6%)
[55 ; 65[	738 (9.9%)
[65 ; 75[	2,001 (26.8%)
≥ 75	4,409 (59.1%)
**Non-ocular comorbidities, n (%)**	
Diabetes	1,390 (18.6%)
Malignant neoplasm	1,041 (14.0%)
Cardiovascular disease	5,576 (74.7%)
Ocular comorbidities, n (%)*	
Diabetic retinopathy	49 (0.7%)
Retinal vein occlusion	2 (0.0%)
Uveitis	1 (0.0%)
Other ocular comorbidities, n (%)**	
OHT / Glaucoma	1,049 (14.1%)
Treatment for DME, ARMD, RVO	277 (3.7%)
Retinal Detachment	44 (0.6%)
Vitrectomy	19 (0.3%)
Previous ophthalmological procedures, n (%)*	
Cataract surgery	2,466 (33.0%)
Nd:YAG posterior capsulotomy	225 (3.0%)

*: assessed over a 2-year period prior to index date; **: assessed over a 1-year period prior to index date

ARMD: age-related macular degeneration, DME: diabetic macular edema, RVO: retinal vein occlusion

About 15% of patients had a history of diabetes (n = 1,390, 18.6%) or malignant neoplasm (n = 1,041, 14.0%). Almost 75% of patients (n = 5,576, 74.5%) had a cardiovascular disease. Ocular comorbidities were identified in 35.6% (n = 2,654) of patients. Treated diabetic retinopathy accounted for fewer than 2% of the patients with ocular comorbidities (n = 49), while RVO and uveitis were almost never encountered. Among known *Nd:YAG-caps* complications, 1,049 (14.1%) patients had a history of OHT/glaucoma, 277 (3.7%) patients received a treatment for either ME, ARMD or RVO, and < 1% of patients had a history of RD (n = 44, 0.6%) or vitrectomy (n = 19, 0.3%).

### Time to nd:YAG posterior capsulotomy

Among the 7,462 patients included, 2,466 (33.0%) had a cataract surgery 2 years before *Nd:YAG-caps*, of whom 732 (29.7%) were performed within the year before the index date while 1734 (70.3%) surgeries were performed between one and two years before it. More than half of them (n = 1,354, 54.9%) had two cataract surgeries within 2 years before *Nd:YAG-caps*, with a median (Q1 – Q3) delay between surgeries of 21.0 (7.0–49.0) days. Logistic regression model showed that patients who had a very early *Nd:YAG-caps* (i.e. <1 year post-surgery) were younger (OR 0.721 [0.673–0.772] for a 10-year increase) than those having a late one. They also had a more frequent history of glaucoma (OR 1.516 [1.240–1.855]) or other treated ocular diseases (OR 2.224 [1.610–3.072]). Moreover, patients who had a non-late *Nd:YAG-caps* (within 2 years) were younger than those having a later one. The odds ratio for a 10-year increase were 0.794 [0.757–0.833] and 0.587 [0.454–0.759] either without or with a history of treated ocular disease, respectively. Age and history of treated ocular disease had a significant interaction, making it impossible to analyze these covariates independently. The patients with non-late *Nd:YAG-caps* also had a more frequent history of treated ocular disease compared to those with late *Nd:YAG-caps* (Table 3). Each of the models analyzed in this study showed areas under curve of ≈ 0.60 and Hosmer Lemeshow test p-value of < 0.001, implying that other variables could impact these models.

**Table 3:** Patient Characteristics at *Nd:YAG-caps* Procedure (N = 7,462)

Characteristics		p-value	OR	CI 95%
Very early *Nd:YAG-caps*	Age at index date (per 10 years)	< 0.0001	0.721	[0.673–0.772]
	History of glaucoma	< 0.0001	1.516	[1.240–1.855]
	History of treated ocular disorder*	< 0.0001	2.224	[1.610–3.072]
Non-late *Nd:YAG-caps*	Age at index date (per 10 years)			
	Without history of treated ocular disorder*	< 0.0001	0.794	[0.757–0.833]
	With history of treated ocular disorder*	< 0.0001	0.587	[0.454–0.759]
	History of treated ocular disorder* (yes/no)			
	At 40 years of age	0.0232	4.894	[1.793–13.357]
	At 50 years of age		3.618	[1.700–7.696]
	At 60 years of age		2.674	[1.594–4.486]
	At 70 years of age		1.977	[1.437–2.719]
	At 90 years of age		1.461	[1.118–1.910]
	History of Glaucoma	0.0213	1.178	[1.025–1.354]

*ocular disorder includes: diabetic macular edema, age-related macular degeneration, and retinal vein occlusion

**Fig. 3:**
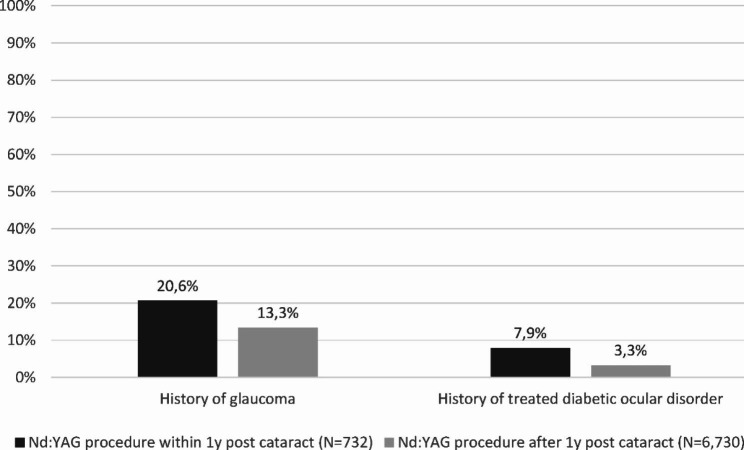
Proportion of patients with a history of glaucoma or treated diabetic ocular disorder among patients with *Nd:YAG-caps* procedure within or after 1-year post cataract surgery (N = 7,462)

The impact of young age on time to *Nd:YAG-caps* was highlighted by the proportion of patients aged < 65 years in each subgroup. This proportion was 22.4% among patients with very early *Nd:YAG-caps*. It decreased to 13.7% and 13.0% among patients with early and late *Nd:YAG-caps*, respectively. A similar trend was observed for patients with a history of glaucoma, as proportions decreased from 20.6 to 13.8% and 13.2%, for the same periods (Fig. 3).

## Discussion

Our study estimated the epidemiology and the characteristics of patients undergoing *Nd:YAG-caps*, using an innovative approach taking *Nd:YAG-caps* as a starting point and analyzing prior cataract surgeries in a backward way.

### Demographics

The mean age at *Nd:YAG-caps* was in line with the literature with mean ages at cataract surgery being between 74 and 77 years [5, 20, 21]. The proportion of women was 64.6% in this study, also in line with Daien et al’s study, with 59% of the cataract surgeries being among women, while the National Ophthalmology Database (NOD) audit from United Kingdoms showed that women seemed slightly more at risk of developing PCO (NOD audit-appendix 8) [5, 20].

According to the EGB data extrapolation, ≈ 253.10^3^ patients have undergone *Nd:YAG-caps* in 2017, accounting for ≈ 310.10^3^ procedures. During the same year, the French national health insurance database for reimbursed procedures (Open-CCAM) recorded 277.10^3^*Nd:YAG-caps* [22]. As Open-CCAM does not exhaustively includes *Nd:YAG-caps* performed among outpatients in public hospitals, it seems acceptable to assess that 290.10^3^ to 300.10^3^*Nd:YAG-caps* are performed each year in France. The geographical distribution of *Nd:YAG-caps* seemed unsurprisingly linked to that of cataract surgeries, with the South being the most frequent region for cataracts and *Nd:YAG-caps*, as described in the 2019 French health authority report [23]. As shown in supplementary material displaying the geographic distribution of *Nd:YAG-caps*, cataract surgeries, and the density of ophthalmologists in France for the year 2017, these 3 components are highly correlated. Over our study’s 4 years (2014–2017), the increase in capsulotomies (+ 19.1%) appeared to have been slightly greater than the increase in cataract surgeries (+ 11.5%) [24]. It cannot be ruled out that the postoperative visual expectations of patients have increased over time, with an earlier request for capsulotomies.

### Clinical characteristics and medical history

The proportion of patients with a history of diabetes was 18.6%, close to that from the French public health organism *Santé Publique France* in 2013, with around 16% of diabetic patients among subjects aged ≥ 70 years [25]. The most frequent comorbidities were cardiovascular impairments (75%). This is in line with the literature and might be mainly represented by high blood pressure (HBP). According to Yazdanyar et al. 2009 study, the prevalence of cardiovascular diseases in the United States was between 70% and 85% among patients aged ≥ 60 years [26]. The global burden of disease project estimated that non-HBP cardiovascular disease prevalence was around 43% among patients aged ≥ 75 years, while Esteban’s study estimated HBP prevalence in France at almost 70% among patients aged between 65 and 74 years [27, 28]. Hence, controlled HBP might not be of major risk for cataract or *Nd:YAG-caps*, despite being captured in this study.

In this study, 14.1% of patients had a history of OHT/glaucoma, in line with Delcourt et al. study from 2010, with a prevalence [CI 95%] of treated OHT and Glaucoma among elderly patients being 9.8 [6.7%; 12.9%] and 5.3% [3.0% ; 7.6%], respectively [29]. Similarly, OHT rates were estimated at 15.5% and 7.5% among men and women aged ≥ 60 years, respectively, by Bron et al. in 2006 [30].

This study is the first to highlight a potential link between several ocular pathologies and earlier *Nd:YAG-caps*. Despite young age and history of uveitis being risk factors already identified in daily practice, macular diseases and glaucoma were not known as such. A recent study based on 2008 to 2018 SNDS data showed that diabetic maculopathies and retinopathies were encountered among 0.1% and 0.2% of the overall French population, respectively. In the same study, the prevalences of treated DME and ARMD were about 0.1% and 1.0%, respectively [31]. In our study, the proportion of severe diabetic retinopathy reached 0.7%, while treated DME, ARMD, and RVO represented almost 4% of the patients. It seems that patients with an active macular disease were over-represented in *Nd:YAG-caps* population when compared to the overall French population. In spite of the greater frequencies of these pathologies observed in patients with earlier Nd:YAG-caps, a causal link with PCO remains unproven. Indeed, patients with chronic ocular disorders might simply have more regular scheduled ophthalmological visits. Hence, *Nd:YAG-caps* might be performed as soon as PCO is observed and before significant visual symptoms are recorded.

### Time to nd:YAG posterior capsulotomy

More than half of patients with a *Nd:YAG-caps* within 2 years post-surgery had both eyes operated on, with an interval of 21 days, in line with Daien et al. EPISAFE study, with 29 days [20]. In the same study, cataract was performed on both eyes among 51.2% of patients, which seemed also in line with our results, even though surgery laterality cannot be assessed in EGB. In this study, most patients had *Nd:YAG-caps* > 2 years after cataract surgery, with 33.0% of patients having *Nd:YAG-caps* procedures performed within 2 years. It is comparable to the French observational studies from Ton Van et al. and Bourdiol Ducasse et al., which showed a mean time to *Nd:YAG-caps* of 32.17 months and around 25 months, respectively, in smaller cohorts [32, 33].

Unsurprisingly, elderly patients appeared to be less likely to have non-late *Nd:YAG-caps* than younger ones. A similar pattern was highlighted by Miller et al. US cohort which showed that older age was a protective factor for early *Nd:YAG-caps* (OR: 0.95 [0.92; 0.98]) [34]. On the other hand, patients with ocular comorbidities seemed more likely to undergo *Nd:YAG-caps* earlier than those without, potentially due to a closer ophthalmological follow-up, allowing an earlier detection of PCO. As models in this study did not exhaustively include all factors, this study cannot be used to assess whether a specific group of IOL results in earlier or more frequent capsulotomies, nor to assess *Nd:YAG-caps-free* survival. The NOD study brought substantial information on the probability of *Nd:YAG-caps* and the time between cataract and *Nd:YAG-caps* according to the type of IOL used. In fact, it showed that PCO rates can widely vary with material and design, at equivalent time points. According to the IOL material used (e.g. hydrophobic or hydrophilic IOL), the 2-year proportion of patients with subsequent *Nd:YAG-caps* varied from 2.2 to 7.0% and from 9.2 to 25.4% after 5 years, showing a threefold increase of *Nd:YAG-caps* procedures with hydrophilic IOLs [5].

### Strengths and limitations

Overall, the French medico-administrative databases do not allow to control the data validity and quality. Some studies have reported that diagnoses coded in the French hospitalization database (PMSI) are not always reliable, leading to a risk of information bias related to coding errors. However, considering the large number of patients included, this bias is expected to have a limited impact. Similarly, it is not possible to ensure the completeness of the SNDS data, particularly regarding comorbidities. To improve data quality, algorithms combining inpatient and outpatient data (diagnoses, reimbursed drugs…) are an effective solution to optimize the identification of pathologies within the SNDS. The study results have been compared to the literature when available. The algorithms used in this study were reviewed by an independent scientific committee and are adapted from validated sources such as French national health insurance (Cnam) mapping of diseases, and literature [35]. A laterality bias can also be highlighted, as no specific clinical data is available in EGB to distinguish which eye has been operated on. However, an important proportion of patients seemed to undergo bilateral cataract surgery, as shown in the Schweitzer et al. FEMCAT study in France, with 63% of patients undergoing bilateral cataract surgery within a short interval. These findings mitigate the importance of the laterality bias [21]. Finally, the medico-administrative nature of claims databases such as EGB leads to a lack of clinical information, limiting the granularity of analyses undergone. For instance, no data is available on the surgeon performing the cataract surgery, the technique used per se, or the type of IOL implanted, which is billed as part of the diagnosis-related group. As specified above, and as shown in NOD audit, these factors can change the outcomes of a surgery, notably in terms of PCO rate [5]. However, the FEMCAT economic study showed that FLACS technique remains rarely used due to a relative lack of medico-economic advantage; and Monnet et al. multicentric analysis showed that multifocal implants represented around 6% of the IOLs in 2021 [36, 37]. These results tend to limit the potential biases due to the differences in practices.

Also, patients with ocular comorbidities could schedule more frequent visits with their ophthalmologists and may benefit from an earlier detection and *Nd:YAG-caps* for PCO. Similarly, health literacy can increase patients’ sensitivity to cataract-related ocular complications and lead to more frequent ophthalmologist appointments on patient’s initiative. Lin et al. Chinese cross-sectional study assessed the link between health literacy and patient-physician communication among patients with cataract, showing a threefold increased probability of poor patient-physician communication if inadequate health literacy (OR 3.6 [1.6; 8.1]) [38].

The main strength identified in this study is the source of data used. EGB is a nationwide population-representative sample of French health insurance beneficiaries, exhaustively gathering every reimbursed healthcare resource used by patients. When considering frequent diseases or procedures, as is the case with *Nd:YAG-caps*, the EGB brings a very high level of evidence, strong external validity, and good data generalizability, reinforced by the availability of a specific *Nd:YAG-caps* code. This study gave a good and informative estimate of the current use of *Nd:YAG-caps* in France, as well as an overview of the profiles of patients undergoing this procedure. Furthermore, the association of results from national claims database with that of clinical studies focused on the ophthalmological risk factors of PCO (e.g., type of IOL, use of coaxial I/A…) could provide important information to the health authorities on which techniques and technologies to promote in order to decrease the number of PCO [5, 39].

## Conclusion

This study allowed us to fill the informational gap regarding real-world data about *Nd:YAG-caps* and give an updated insight into patients’ characteristics and main comorbidities. This study estimated that more than 250.10^3^ patients undergo *Nd:YAG-caps* each year in France, accounting for more than 300.10^3^ procedures in 2017. More than 30% of *Nd:YAG-caps* were performed within 2 years after the cataract surgery. When analyzing the patients’ characteristics at the date of *Nd:YAG-caps*, it appeared that patients with a history of ocular disease were more likely to undergo non-late *Nd:YAG-caps*. In the meantime, despite cataract surgery being mainly performed among older patients, younger ones seemed to have a shorter period between cataract and *Nd:YAG-caps*. This data must be considered as refractive cataract procedures are mostly undergone among younger patients.

### Electronic supplementary material

Below is the link to the electronic supplementary material.

Supplementary Material 1

## Data Availability

Complete list of algorithms is available as Supplementary data. No additional data is available.
