# Supplementary material for: Incidence of Nd:YAG laser capsulotomy following cataract surgery: a population-based nation-wide study – FreYAG1 study
Source: BMC Ophthalmol. 2023 Oct 16;23:417. doi: 10.1186/s12886-023-03134-6 (PMC10578013; doi:10.1186/s12886-023-03134-6)
Supplement: Supplementary file 1 — Supplementary Material 1 [file 12886_2023_3134_MOESM1_ESM.docx]

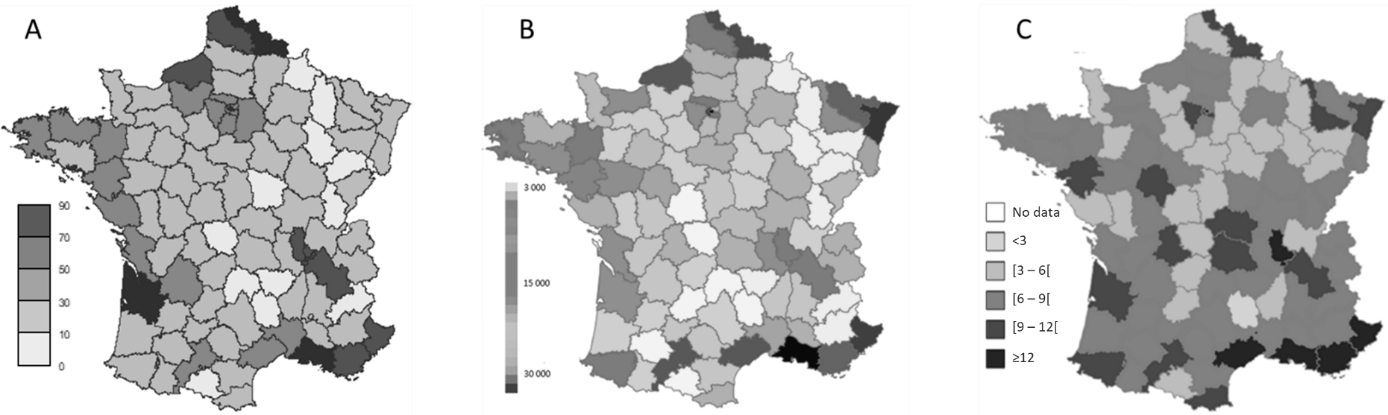


Figure 1 suppl: **French Geographic Repartition of *Nd:YAG-caps* Procedures (A – source: FreYAG1 study), Cataract Surgeries (B – source: France Opendata OpenCCAM), and French ophthalmologist density for 100,000 inhabitants (C – source: *Cour des Comptes* report October 2018) for the year 2017**

# Abbreviations

| ATC | Anatomical, Therapeutic and Chemical classification |
| --- | --- |
| CCAM | French procedure classification (Classification Commune des Actes Médicaux) |
| CIP | Drug packaging code (Code identifiant de Présentation) |
| ICD-10 | International Classification of Diseases – 10th revision |
| LTD | Long Term Disease |
| MCO | Medicine, Surgery, Obstetrics settings (Médecine Chirurgie Obstétrique) |
| PMSI | National Hospitalization database (Programme de Médicalisation des Systèmes d’Information) |

# Population Selection

## Inclusion Criteria

The study population will include patients :

- With continuous insurance coverage over data extraction period;
- Who had undergone a posterior capsulotomy for secondary cataract with Nd:YAG laser (procedure code [CCAM] BFPP001) between January 1^st^ 2014 et December 31^st^ 2017;
- Aged 18 years and older at the time of Nd:YAG capsulotomy.

## Exclusion criteria

None

# Medical and Treatment History

Medical and treatment history will be assessed **over a 2-year period prior to index date**.

| Criteria | Codes & algorithms |
| --- | --- |
| **Non-ocular comorbidities** | |
| Diabetes | - ≥1 inpatient stay (PMSI MCO) or LTD with **ICD-10 codes E10 to E14**   AND/OR   - ≥ 3 reimbursements at different dates for antidiabetics with **ATC codes A10A or A10B** |
| Malignant neoplasm | - ≥1 inpatient stay (PMSI MCO) or LTD with **ICD-10 codes C00 to C97** |
| Cardiovascular disease | - ≥1 inpatient stay (PMSI MCO) or LTD with **ICD-10 codes I00 to I99 or Q21**   AND/OR   - ≥ 3 reimbursements at different dates for a cardiovascular drugs with **ATC codes C01, C02, C03, C04, C05, C08, C09, C10** |
| **Ocular comorbidities** | |
| Diabetic retinopathy | - ≥1 inpatient stay (PMSI MCO) with **ICD-10 codes H36.0, E10.3 or E14.3** |
| Retinal venous occlusion | - ≥1 inpatient stay (PMSI MCO) with **ICD-10 codes H34.8 or H34.9** |
| Uveitis | - ≥1 inpatient stay (PMSI MCO) with **ICD-10 codes H20.x** |
| **Other ocular comorbidities** | |
| OHT/glaucoma | - ≥1 inpatient stay (PMSI MCO) or LTD with **ICD-10 codes H40.0 to H40.9**   AND/OR   - ≥3 reimbursements for anti-glaucoma treatment, with **ATC code S01E** (except carbachol S01EB02)   AND/OR   - ≥1 laser procedure for glaucoma with **CCAM code BENP001**   AND/OR   - ≥1 surgical procedure for glaucoma with **CCAM codes BEFA008, BEJB004, BENA002, BEPA002, BEPA003, BEPB001, BGFA014, BGFA900** |
| Treated macular edema, age-related macular degeneration, retinal venous occlusion | - ≥1 reimbursement for dexamethasone implant with **CIP code 3400949407118**   AND/OR   - ≥2 consecutive reimbursements on a 2-month period for acetazolamide with **CIP code 3400930305270**   AND/OR   - ≥2 reimbursements for anti-VEGF drugs with   - aflibercept **ATC code S01LA05**   - ranibizumab **ATC code S01LA04**   - bevacizumab     - **CIP codes 3400937810159**     - **UCD codes 9261104, 9261110, 9876544**   AND/OR   - ≥1 procedure for intravitreal injection with **CCAM code BGLB001**   AND/OR   - ≥1 inpatient stay (PMSI MCO) with **ICD-10 code H20.x** AND ≥1 reimbursement for steroid eyedrop (**ATC code S03BA**) or systemic steroid (**ATC codes H02A and H02B**) OR ≥1 therapeutic ocular injection with **CCAM code BKLB001**   AND/OR   - ≥1 inpatient stay (PMSI MCO) with ICD-10 code H19.0 AND ≥ 2 reimbursements for anti-infectives (**ATC codes J01, J04A or J05**)   AND/OR   - ≥1 inpatient stay (PMSI MCO) with ICD-10 code H22.1 AND ≥ 2 reimbursements for systemic steroids (**ATC codes H02A and H02B**) or immunosuppressants (**ATC codes L04A**)   AND/OR   - ≥1 inpatient stay (PMSI MCO) with ICD-10 code H22.0 AND ≥ 2 reimbursements for anti-infectives (**ATC codes J01, J04A or J05**) |
| Retinal detachment | - ≥1 inpatient stay (PMSI MCO) with **ICD-10 code H33 or H35.7**   AND/OR   - ≥1 procedure for retinal detachment management with **CCAM codes BGBA001, BGDA001, BGDA002, BGDA003, BGDA004, BGDA006, BGDA007, BGDA008, BGMA003, BGFA006, BGFA009 , BGFA010** |
| Vitrectomy | - ≥1 **CCAM code BGFA001, BGFA005, BGFA006, BGFA008, BGFA009, BGFA010, BGFA011** |
| **Previous ophthalmological procedures** | |
| Capsulotomy | - ≥1 record for Nd:YAG posterior capsulotomy with **CCAM code BFPP001** |
| Cataract surgery | - ≥1 record for cataract surgery with artificial lens implantation, with **CCAM codes BFGA002, BFGA004, BFGA006**   AND/OR   - ≥1 record for cataract surgery without artificial lens implantation, with **CCAM codes BFGA008, BFGA009, BFGA010**   - Associated with a lens implantation procedure with **CCAM codes BFLA001, BFLA003, BFLA004** |
